# Primary Cardiac Synovial Sarcoma: A Case Report and Brief Review of the Literature

**DOI:** 10.1155/2007/94797

**Published:** 2007-07-05

**Authors:** Brian Boulmay, Gary Cooper, John D. Reith, Robert Marsh

**Affiliations:** ^1^Division of Hematology/Oncology, Department of Medicine, Health Science Center, University of Florida, P.O. Box 100277, Gainesville, FL 32610, USA; ^2^Division of Cardiology, Department of Medicine, Health Science Center, University of Florida, P.O. Box 100277, Gainesville, FL 32610, USA; ^3^Department of Pathology, Health Science Center, University of Florida, P.O. Box 100277, Gainesville, FL 32610, USA

## Abstract

Synovial sarcoma comprises approximately 10% of all soft tissue sarcoma diagnoses; a primary synovial sarcoma of the myocardium is exceedingly rare. There have been very few cases reported in the literature thus far. With the identification of the characteristic and diagnostic chromosomal abnormality t(X;18), this may become an increasingly recognized entity. Our report adds to the limited published cases of primary cardiac synovial sarcoma with the characteristic t(X;18). Further elucidation of the effects of this translocation on the cell cycle may lead to directed therapies in the future.

## 1. INTRODUCTION

Synovial sarcoma is an uncommon malignancy, comprising approximately 10% of all soft-tissue sarcomas (STS) [1]. Unlike other STS, the synovial type occurs most often in children and young adults and is an aggressive tumor with 10-year-old survival rates reported in some series as low as 0–20% [2, 3]. It is divided into three subtypes: biphasic, monophasic and poorly differentiated. Historically these nonspecific histologic characteristics have made the establishment of a definitive diagnosis problematic. However, a characteristic chromosomal abnormality for synovial sarcoma has been identified for all morphologic subtypes: t(X;18) [4].

Primary cardiac synovial sarcoma with the characteristic t(X;18) has rarely been reported in the medical literature (see Table 1) [5–10]. We report a recent case from Shands Hospital at the University of Florida.

## 2. CASE REPORT

The patient is a 19-year-old woman who had complaints consisting of shortness of breath, vague chest pain, and increasing exercise intolerance over three months. One week prior to admission, she noted intermittent palpitations and the onset of new bilateral lower extremity edema. Physical examination was significant for a 2/6 pansystolic murmur heard at the right lower sternal border and trace pitting lower extremity edema. Continuous ECG monitoring demonstrated intermittent four to five beat runs of ventricular tachycardia.

A computed tomography scan of the chest and abdomen showed an enlarged heart with a lobulated mass in the right ventricle. A right-sided pleural effusion, two enlarged internal mammary lymph nodes, and multiple scattered bilateral pulmonary nodules were also noted. A two dimensional echocardiogram with doppler confirmed that the right ventricle was essentially filled with mass. A second mass was noted to arise from the tricuspid annulus causing severe tricuspid regurgitation and obstructing right ventricular inflow.

**Figure 1:**
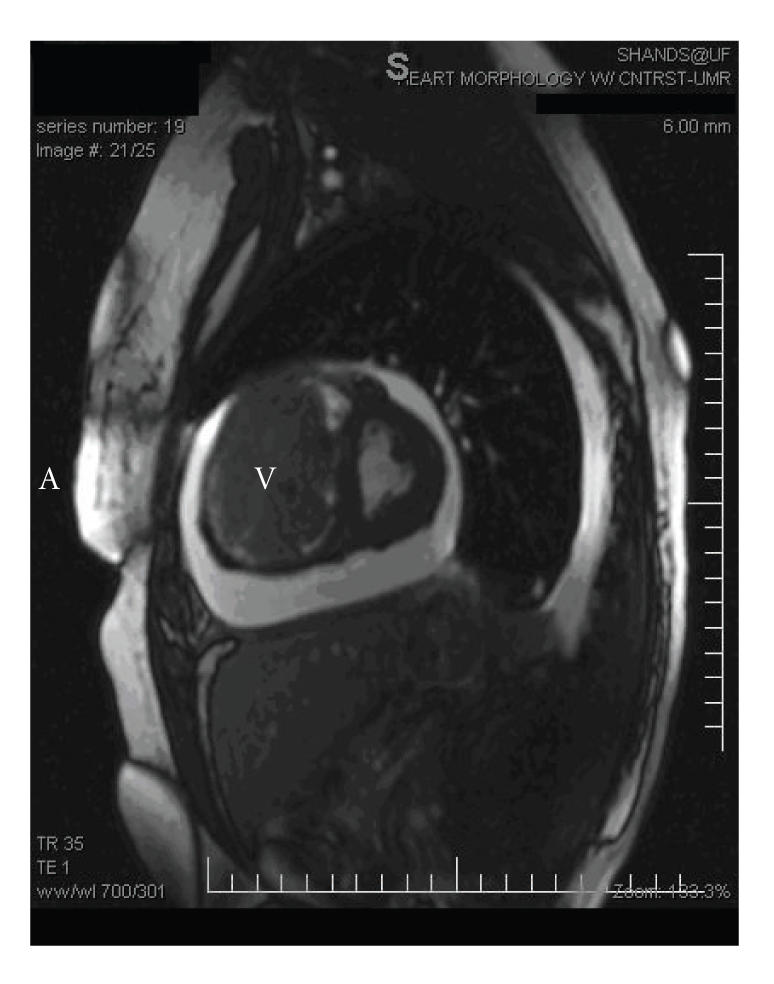
Short axis view shows a large tumor filling almost the entire right ventricle (V).

**Figure 2:**
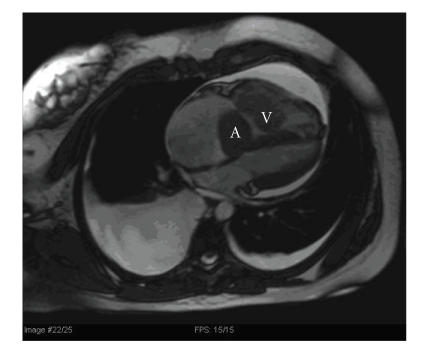
White blood 4 chamber view shows bilateral pleural effusions, pericardial effusion, right atrial and right ventricular enlargement. There is a multilobed tumor mass in the right atrium (A) and right ventricle (V).

**Figure 3:**
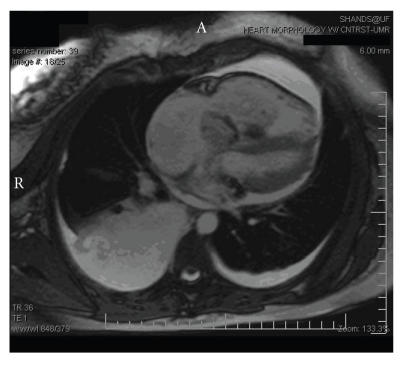
Post-gadolinium infusion delayed enhancement study reveals significant uptake of gadolinium consistent with edema, fibrosis or scar.

The decision was made to perform a definitive resection, both for diagnosis and restoration of hemodynamic stability. Preoperatively, cardiac magnetic resonance imaging demonstrated a bilobed mass within the heart (see Figures 1, 2 and 3). One mass was found to fill the right ventricle with extension into the right ventricular outflow tract and pulmonary artery. Also confirmed was a mass involving the tricuspid valve, resulting in marked regurgitation with right atrial and inferior vena cava distension.

## 3. OPERATIVE FINDINGS

Sternotomy was performed and as the right hemithorax was entered, multiple nodules were noted on the visceral and parietal pleural surfaces. A right middle lobe and hilar lesion were resected. The heart was inspected and tumor was seen through the epicardial surface between the right atrium and aorta. After entering the right atrium, tumor was noted to be growing from the tricuspid annulus and this was resected down to the right atrial wall. A 2 × 2.5 cm portion of tumor was left on the atrial septum to preserve tricuspid function. Next, the pulmonary artery was entered and tumor was resected back through the pulmonic valve to the level of the myocardium. A small portion of tumor was left at the crista terminalis measuring approximately 1.5 cm.

## 4. PATHOLOGIC FINDINGS

The intraoperative frozen section from both lung and cardiac masses showed a “high-grade sarcoma.” On definitive pathologic examination, the tumor appeared to have features of a high-grade synovial sarcoma. Immunohistochemical staining showed that the tumor was CD99 and BCL-2 positive further suggesting that this was a synovial sarcoma. Cytogenetics were obtained which showed the following karyotype: 49, X, t(X;18)(p11.2;q11.2), +4, +7, +8, der(13;15)(q10:q10), +14(20). The t(X;18)(p11.2;q11.2) confirmed the diagnosis of synovial sarcoma.

## 5. CLINICAL OUTCOME

The patient was discharged on post-operative day nine with no complication. She had experienced marked improvement in her shortness of breath. She elected not to pursue palliative chemotherapy and at four months of follow-up retained good functional status with no symptoms.

## 6. DISCUSSION

The designation “synovial” sarcoma implies origin from normal synovium. As alluded to previously, it is histologically described as having a biphasic appearance consisting of two distinct morphologic subtypes: spindle and epitheliod cell subtypes. Interestingly, neither epithelial differentiation nor IHC staining for epithelial markers like cytokeratin are found in normal synovium. The true tissue of origin is, therefore, still unknown. Synovial sarcoma occurs primarily in the extremities and is associated with the large joints, such as the knee. Contrary to what would be expected, these tumors are rarely present inside the joint capsule. Instead, they are found most often in association with bursae and tendon sheaths. Synovial sarcomas can also be found in structures without a joint capsule such as in the head and neck and in lung [11]. Primary cardiac synovial sarcomas are exceedingly rare

Primary cardiac sarcomas of all types have poor outcomes; primary cardiac synovial sarcoma is no different, with a mean survival of 9 to 16.5 months [12]. In the cases of synovial sarcoma for which outcomes are reported, all but one patient died within 1 year. A possible exception to this uniformly poor prognosis has been reported by Sassani et al. [12]. They describe the case of a 45-year-old man with a right atrial mass who had complete surgical resection. The tumor was a biphasic synovial sarcoma, however, confirmatory cytogenetics could not be performed due to fixative technique. No adjuvant chemotherapy was given and at 5 years the patient was reported to be alive [12].

The characteristic chromosomal abnormality of synovial sarcoma is t(X;18)(p11.2;q11.2) and is present in 90–100% cases, suggesting strongly that it is directly involved in some aspect of the malignant process [13, 14]. The t(X;18) fuses two genes, SYT at 18q11 and SSX at Xp11. The protein products of both of these two genes are transcription regulators present in the cell nucleus. The normal SSX codes for a protein that inhibits transcription; SYT on the other hand encodes a protein that acts as a transcriptional activator [15]. The t(X;18) results in a replacement of the inhibitor region of SSX with the activator portion of SYT. Although the genes normally repressed by SSX are not well described, the fusion protein SYT/SSX likely acts as a transcription derepressor [14]. SYT/SSX is also thought to regulate chromatin remodeling which may lead to enhanced proliferation of mesenchymal cells [16].

Standard therapy for synovial sarcoma classically involves cytotoxic therapy. As compared to other soft tissue sarcomas, the synovial subtype is relatively chemosensitive; response rates as high as 58% are reported in some series [17]. Ifosfamide and adriamycin have shown the most consistent efficacy in synovial sarcoma of all chemotherapy drugs used in soft tissue sarcomas, including gemcitabine, docetaxel, bortezomib, imantinib, and others [18]. However, both of these agents are toxic and in the metastatic setting there is no standard therapy that is clearly superior to others. The need for novel therapeutic agents in this disease is clear and the translocation t(X;18) with the resulting SYT/SSX fusion protein makes synovial sarcoma an ideal candidate for molecular targeting. The ambiguous function of the fusion protein leaves much to be done before development of targeted therapy is completed. Some work has been done using immunotherapy to target the SYT/SSX fusion protein. Patients with synovial sarcoma often have cytotoxic lymphocytes specific for the SYT/SSX protein [19]. A phase I study using an SYT/SSX derived junction peptide vaccine in six patients with synovial sarcoma has demonstrated some efficacy [20].

The case that we present is unusual in that in addition to t(X;18) there are five more cytogenetic abnormalities. Only two cases in the literature of cardiac synovial sarcoma with a reported t(X;18) have had complex abnormalities; the first involved chromosome 7 [6] and the second reported a complex karyotype [5]. In cases of noncardiac synovial sarcomas, the majority have a diploid or near diploid karyotype. Retrospective data have shown that patients with synovial sarcoma and complex chromosomal abnormalities have a greater tendency to metastasize; this is consistent with the view that with tumor progression comes additional genetic abberations [13]. As more primary cardiac synovial sarcomas are reported perhaps these tumors will be found more likely to have complex cytogenetics as compared to the extremity type.

## 7. CONCLUSION

We have reported a patient with primary cardiac synovial sarcoma presenting in the right atrium and ventricle with the characteristic t(X;18)(p11.2;q11.2) and additional cytogenetic abnormalities. This complex karyotype has previously been reported twice in the literature. Synovial sarcoma of the heart is extremely rare and typically not included in the differential diagnosis of cardiac masses. Often the diagnosis may be missed due to the nonspecific histologic and immunohistochemical characteristics of a tumor which often may be labeled as a “poorly differentiated” sarcoma. The clinical outcomes are universally poor despite aggressive therapies. The detection of t(X;18)(p11.2;q11.2) can be a useful adjunct to the work-up of a cardiac sarcoma. The therapeutic implications of establishing a firm diagnosis may be important in the future if targeted therapies become available.

## Figures and Tables

**Table 1:** 

Author	Age	Sex	Cytogenetics	Treatment	Site of tumor	Follow-up

Karn [7]	35	M	t(X;18)	Surgery and chemotherapy	RA and pericardium	Died at 9 months
Iyengar [6]	38	M	t(X;18)der7	Surgery	RV	Died at 1 year
Oizumi [10]	19	F	t(X;18)	Surgery	Pericardium	Died at 7 months
Al-Rajhi [9]	19	M	t(X;18)	RT	Pericardium	Alive at 12 months
McGilbray [8]	30	M	t(X;18)	Surgery and chemotherapy	Mitral valve	N.A.
			61,Y,der(X)t(X;18)(p1;p1)x2,			
			der(1)t(1;8)(q10;q10),			
			der(4)t(4;14)(p14;q11∼12),			
			+5,−6,del(7)(p13∼14),			
			−9,−10,−11,			
			der(11)t(11;12)(q10;p10)x2,			
Hazelbag [5]	42	M	der(12)t(X;12)(p;q),−13,	Surgery	LA/LV, lung/liver mets	Died at 1 month
			−14,−16,+17,−18,			
			del(18)(p10),−20,−21,			
			der(22)t(12;22)(q12;q12)t(11;			
			12)(q24∼25;q24),			
			der(22)t(5;22)(p10;			
			p11)x2[cp25]			
			49,X,t(X;18)(p11.2;q11.2),			
Current case	19	F	+4,+7,+8,der(13;15)(q10:q10),	Surgery	RA/RV, lung metastasis	Alive at 4 months
			+14(20)			
